# Interplay of Auxin and Cytokinin in Lateral Root Development

**DOI:** 10.3390/ijms20030486

**Published:** 2019-01-23

**Authors:** Hongwei Jing, Lucia C. Strader

**Affiliations:** Department of Biology, Washington University, St. Louis, MO 63130, USA; hjing@wustl.edu

**Keywords:** lateral root, auxin, cytokinin, crosstalk

## Abstract

The spacing and distribution of lateral roots are critical determinants of plant root system architecture. In addition to providing anchorage, lateral roots explore the soil to acquire water and nutrients. Over the past several decades, we have deepened our understanding of the regulatory mechanisms governing lateral root formation and development. In this review, we summarize these recent advances and provide an overview of how auxin and cytokinin coordinate the regulation of lateral root formation and development.

## 1. Introduction

Roots are crucial for the perception and uptake of water and nutrients, anchorage of the plant body, storage of nutrients, and vegetative reproduction [1]. Root systems consist of two principal root types: the primary root, which is formed embryonically, and secondary roots, which form post-embryonically [2]. These secondary roots encompass both lateral roots (LRs), formed as branches from the primary root, and adventitious roots, developed from shoots, stems, or leaves [2,3]. Root system architecture is determined by root growth, root angle and branching through lateral root formation [4]. Lateral root formation and development play vital roles in driving plant root system architecture. As a branched root system, lateral roots also provide a mechanism for the plant to explore the soil to obtain water and nutrients as well as providing anchorage [5]. At present, lateral root initiation and development have been well characterized in the herbaceous model plant *Arabidopsis thaliana*. In Arabidopsis, LRs originate from pericycle cells adjacent to xylem pole cells. Starting from the first cell division in the pericycle cell up to the emerging LR primordium, seven stages have been defined that correspond to different steps in the acquisition of cell identity and tissue organization [3,6].

As a potent regulator of root development, auxin plays important roles to control the development and architecture of the root system [2,7]. Auxin biosynthesis, polar transport, and signal transduction are key processes in the promotion of lateral root development [8]. The phytohormone cytokinin is another central regulator of lateral root development [9,10,11,12]. Cytokinin antagonizes auxin response by promoting the degradation of pin-formed (PIN) proteins and interfering with polar auxin transport to regulate lateral root development [10,12]. Additional plant hormones directly or indirectly contribute to cell fate decisions and cooperate differentially in lateral root development [13,14]. In this review, we discuss the roles of auxin and cytokinin in distinct stages of lateral root initiation and development, as well as auxin and cytokinin interactions in the regulation of this important process.

## 2. Lateral Root Development Stages

Unlike in animals, in which most organ development occurs during embryogenesis, plant organogenesis primarily takes place post-embryonically. Meristems give rise to post-embryonic cells that constitute different organs throughout a plant’s lifetime. In plants, there are two main meristems: the shoot apical meristem (SAM) and the root apical meristem (RAM) [15]. The Arabidopsis RAM contains a stem-cell niche (SCN) with a central organizer, termed the quiescent center (QC). These stem cells yield all the major tissues that compose the root. The initial stem cells divide asymmetrically into two cells: one of these retains its stem cell identity, whereas the other one divides anticlinally and eventually elongates and differentiates into a specific root cell type. The growing root can be divided into four developmental zones from root tip to shoot: (i) the meristematic or proliferation zone with active cell divisions; (ii) the transition zone with slow cell growth in both length and width; (iii) the elongation zone with rapid growth in cell length but not width; and (iv) the differentiation zone, in which cells cease to expand and start to differentiate into their specialized identities [16,17].

**Figure 1:**
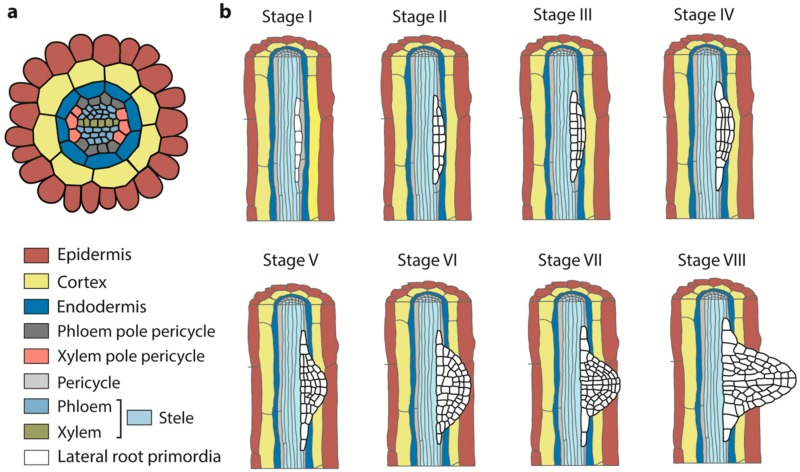
Morphological changes during lateral root (LR) initiation and development. (**a**) Schematic representation of the Arabidopsis primary root cross-section. The root has a simple structure composed of the stele (pericycle and vasculature) surrounded by three one-cell layer, endodermis, cortex and epidermis. (**b**) The eight stages of lateral root initiation and development. Lateral root founder cells (LRFCs, white color in Stage I) are specified in the xylem–pole–pericycle (XPP) and pass through eight developmental stages to emerge at the surface of the root. Stages I–IV take place before the endodermis is traversed and Stages V–VIII take place after the endodermis has been crossed.

The Arabidopsis primary root has a radial structure of concentric cylinders of different cell types, including the epidermis, cortex, endodermis, and stele (pericycle and vasculature) (Figure 1a) [16,18]. The layers of epidermal, cortical, and endodermal tissues surround a single-layered pericycle and central vasculature tissues (Figure 1a) [16,18,19]. The pericycle is a heterogeneous tissue with diarch symmetry composed of two cell types: phloem–pole–pericycle (PPP) cells and xylem–pole–pericycle (XPP) cells (Figure 1a) [16,18,19]. XPP cells are those pericycle cells that are thought to be semi-meristematic and have cell division ability. LR originate exclusively from XPP cells, also called pericycle founder cells (Figure 1a) [20]. LR formation consists of four stages: prebranch site formation, initiation, primordium formation, and emergence (Figure 1b).

### 2.1. LR Prebranch Site Formation

LR prebranch site formation usually occurs in the transition zone and the elongation zone, in which lateral root founder cells (LRFCs) are activated to specify the spatial distribution of lateral root primordia (LRP). Lateral root prebranch sites consist of a pair of XPP cells and are a single cell layer generated in the differentiation zone of the root [6]. These adjacent XPP cells, or LRFCs, will undergo asymmetric cell division to produce a single layered primordium containing up to ten small cells, designated as stage I LRP (Figure 1b) [21].

### 2.2. LR Initiation

LR initiation, cell cycle re-entry and asymmetric cell divisions of adjacent xylem pole pericycle cells result in the formation of the LRP. Prior to the first cell division marking lateral root initiation, the nuclei of LRFCs acquire a rounded morphology, and in the case of an abutting pair of LRFCs, migrate toward the cell wall common to the two cells [22].

### 2.3. LR Primordium Formation

Lateral root primordium formation is divided into several stages according to the number of completely formed cell layers in the primordium (Stages III–VII, Figure 1b) [6]. A sign of lateral root primordium formation onset is the formation of an incipient Stage I LRP. Subsequently, cell growth and rounds of anticlinal, periclinal, and tangential cell divisions are launched to establish a dome-shaped primordium that emerges as a lateral root at the final stage of LR primordium formation [17,23,24].

### 2.4. LR Emergence

LR emergence is an interactive process between LRP and their overlaying tissues to finally form a new LR (Stage VIII, Figure 1b) [17,25]. After the formation of the LRP, this primordium develops and eventually breaks through the endodermis, cortex, and epidermis in order to emerge from the primary root. On its way to emergence, the LRP first must breach the endodermis [26]. During the lateral root emergence process, overlying epidermal cells do not appear to lose volume, but rather are pushed away after the loss of cell-to-cell adherence. By contrast, the Casparian strip network keeps the endodermal cells tightly connected, which forces the endodermis to accommodate growth through dramatic volume losses and requires minimal separation of cell walls for lateral root emergence. Eventually, small holes and/or breaking points allow for a localized opening of the network for primordium passage [5,26]. After LRP emergence from the endodermis, cortical and epidermal cells barely change shape and instead are pushed away by a loss of cell-to-cell adherence [5,27,28].

**Figure 2:**
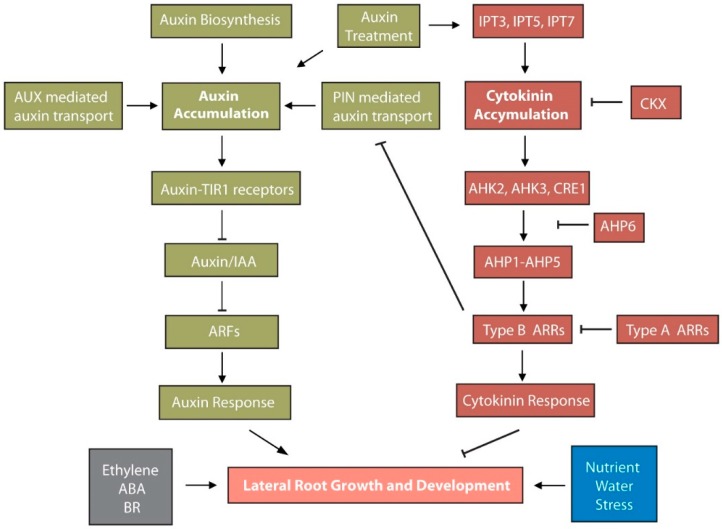
Regulatory interactions between auxin and cytokinin pathways controlling LR development. Auxin acts as a key hormone for LR formation. Components of auxin biosynthesis, transport and signaling including auxin/indole-3-acetic acids (Aux/IAAs) and auxin response factors (ARFs), play important roles in LR initiation and LR primordium development. Cytokinin generally plays an antagonistic role to auxin pathway in LR formation. Cytokinin biosynthesis enzyme IPT, degrading enzyme CKX, and signaling components AHK, ARR, and AHP6, involved in LRs initiation and growth. Arrows and inhibition lines represent positive and negative interactions, respectively. ABA, abscisic acid; AHK, Arabidopsis histidine kinase; AHP, Arabidopsis histidine phosphotransferase; ARR, arabidopsis response regulator; AUX, auxin resistant; Aux/IAA, Auxin/indole-3-acetic acid; ARF, auxin response factor; BR, brassinosteroid; CKX, cytokinin oxidase; CRE1, cytokinin response 1; IPT, isopentenyl transferase; PIN, PIN-formed; TIR1, transport inhibitor response 1.

Recent studies have revealed that phytohormones play distinct and important roles in controlling these lateral root developmental stages. In the next sections, we focus on how the phytohormones auxin and cytokinin, both individually and in combination, regulate lateral root formation and development (Figure 2).

## 3. Roles of Auxin in Lateral Root Development

Auxin plays a central role during lateral root development [4,5,17,20,29,30,31,32]. Here, we review how auxin regulates distinct stages of lateral root initiation and development.

### 3.1. Auxin Regulates Lateral Root Prebranch Site Formation

Lateral root prebranch sites are specified at the transition of the meristem and the elongation zone. These are thought to form by priming specific groups of XPP cells with a distinct molecular identity to confer competence for LR formation. These LR-competent XPP cells are referred to as prebranch sites [21,31]. The priming of pericycle cells takes place in the basal meristem, correlating with elevated auxin signaling in this region [29]. An endogenous mechanism for establishing LR priming was proposed from these data, based on a temporal fluctuation in the expression of the synthetic auxin-response promoter *DR5* [21]. *DR5* promoter output oscillates with the formation of a subsequent lateral root at regular 15 h intervals [29], marking the position of future lateral roots by establishing prebranch sites [33]. The region over which this dynamic expression pattern occurs has been termed the oscillation zone, which is located behind the primary root tip and spans the entire elongation zone [21,33]. In Arabidopsis, lateral roots are spaced along the main axis in a regular left–right alternating pattern that correlates with gravity-induced waving and depends on the auxin influx carrier auxin 1 (AUX1) [29,34]. Moreover, the periodicity of lateral organ induction is correlated with recurrent programmed cell death at the distal edge of the root cap, which appears to release pulses of auxin surrounding root tissues to establish the pattern for LR formation [35]. Overall, these studies suggest that an auxin source in the root tip specifies LRP initiation sites. 

Lateral root prebranch site formation starts with LRFC specification, which determines a subset of competent XPP to initiate LRP [17,21]. Activation of the *DR5* reporter, an indicator of auxin response, is considered the first indication of the acquisition of LRFC identity, suggesting that local activation of auxin response precedes the initiation of LRP formation [36]. The developmental progression of LRFCs to stage I LRPs requires activity of the auxin transporter PIN3 in endodermal cells, which are adjacent to the pericycle cells [37]. However, LRFCs exhibit *DR5:GFP* expression prior to PIN3 accumulation in endodermal cells, suggesting that the LRFC fate has been specified prior to PIN3 accumulation [37]. 

The auxin-regulatory GATA23 transcription factor regulates LRFC identity in the Arabidopsis root [17,22]. *GATA23* is expressed specifically in xylem–pole–pericycle cells prior to the first asymmetric division and is correlated with the oscillating auxin signal in the root tip. Functional studies revealed that an indole-3-acetic acid28 (IAA28)-dependent auxin signaling module in the basal meristem region regulates *GATA23* expression and thereby lateral root founder cell specification and root branching patterns [22]. In addition, *membrane-associated kinase regulator 4* (*MAKR4*) modulates the oscillation signal in the root tip and is required to successfully convert prebranch sites into lateral organs along the primary root [38]. Further, the transcription factor AtMYB93 is expressed strongly, specifically, and transiently in the endodermal cells overlying early lateral root primordia and is induced by auxin in the basal meristem of the primary root [39]. AtMYB93 appears to be a negative regulator of lateral root development in Arabidopsis [39]. Thus, auxin triggers lateral root prebranch site formation and regulates genes essential for LRFC specification.

### 3.2. Auxin Promotes Lateral Root Initiation

Concomitantly with the rounding and migration of LRFC nuclei, LRFCs increase in volume accompanied by the shrinking and deformation of the overlying endodermis [26]. An important auxin signaling module driving LR initiation consists of solitary-root (SLR)/IAA14 and auxin response factor7 (ARF7)/ARF19, in which the auxin-induced degradation of SLR proteins de-represses ARF7 and ARF19 transcriptions factors to activate auxin-related gene expression [40,41,42]. LRP and LRs are virtually absent in both dominant negative *slr* and loss-of-function *arf7arf19* mutant roots [40,41,42]. This signaling module (IAA14-ARF7/19) is considered distinct from the bodenlos (BDL)/IAA12-monopteros (MP)/ARF5 module, which acts after the crucial early SLR/IAA14-ARF7-ARF19–dependent auxin response module [43]. Further, IAA14 degradation is crucial for the first asymmetric divisions that mark the initiation of new root primordia [40,44] in a dynamic and tunable fashion [45]. Thus, IAA14 appears to be a critical auxin-induced reprogrammable timer for lateral root initiation. 

Plants expressing a gain-of-function *short hypocotyl 2* (*shy2-2*) allele in endodermal cells display delayed lateral root emergence [26], suggesting that attenuated auxin response in the endodermis results in the suppression of the progression of lateral root development. Mutations in the rice *lateral rootless 2* (*LRT2*), encoding a cyclophilin-type peptidyl-prolyl *cis/trans* isomerase (PPIase) result in lateral root defects, specifically a block in progression between nuclear migration, but before the first anticlinal division of the pericycle cells [46,47]. Biochemical and genetic studies showed that LRT2 catalyzes the *cis*/*trans* isomerization of rice OsIAA11 at the Trp^104^-Pro^105^ peptide bond to facilitate binding to the auxin receptor OsTIR1 [48]. In addition to regulation of ARF activity through derepression via Aux/IAA degradation, phosphorylation of ARF7 and ARF19 by brassinosteroid-insensitive 2 (BIN2) can also potentiate auxin signaling output during lateral root organogenesis [49]. In conclusion, these results show that key components of auxin signaling transduction play central roles to promote LR initiation. 

### 3.3. Auxin Regulates Lateral Root Emergence

Auxin transport and signaling components play an essential role during LRP emergence [4,17,50,51,52,53]. The auxin influx carriers AUXI and the related LIKE-AUX1-3 (LAX3) facilitate lateral root emergence [50,51]. In particular, LAX3 exhibits a striking expression pattern in cortical cells overlaying a developing LRP; these cells later separate to facilitate organ emergence [51]. LAX3 is necessary for auxin-dependent induction of a set of cell-wall remodeling enzymes which can promote cell separation [51]. Auxin may act as an inductive signal that moves from the inner tissue of the root towards the outer tissue to trigger the LRP emergence response through the sequential induction of PIN3 and LAX3 in these overlying tissues [52,54]. Recent studies found that auxin-inducible LAX3 expression is regulated by the lateral organ boundaries–domain/asymmetric leaves 2-like (LBD/ASL) transcription factor family member LBD29, which itself is a direct target of ARF7 [53]. Disrupting auxin-inducible *LBD29* expression phenocopied the *LAX3* mutant, resulting in delayed lateral root emergence [53], suggesting that sequential *LBD29* and *LAX3* induction by auxin is required to coordinate cell separation to allow lateral root emergence. Overall, these studies indicate that both auxin transport and signal transduction play critical roles to regulate cell loosening to allow lateral root emergence. 

Clearly, auxin regulates every stage of lateral root development, including prebranch sites formation, initiation, and emergence. 

## 4. Cytokinin Roles in Lateral Root Development

The plant hormone cytokinin plays crucial roles in regulating lateral root formation and growth (Figure 2) [9,10,55]. Decades ago, cytokinin was described as having an inhibitory effect on lateral root formation [56,57,58]. Subsequent studies indicate that cytokinin treatment inhibits lateral root initiation and development [59,60,61,62], converse to the stimulatory effects of auxin treatment. Moreover, Arabidopsis mutants defective in cytokinin receptors or in *Arabidopsis response regulators* (*ARRs*) required for cytokinin response display increased root branching [60,61,63]. However, the molecular mechanisms underlying cytokinin effects on lateral root formation have not been fully elucidated, despite recent progress on this front [9,10,12,55].

### 4.1. Cytokinin Disrupts LRFC Formation

Overexpression of the gene encoding the cytokinin-degrading enzyme cytokinin oxidase (CKX) results in reduced cytokinin levels and enhanced root branching [59,64]. Further, Arabidopsis *CKX* genes are expressed in LRPs, suggesting that the removal of the cytokinin signal is important to allowing the progression of lateral root development [64,65]. In support of this possibility, exogenous cytokinin application represses early LR initiation by blocking the transition from the G2 to the M phase in the pericycle founder cells [62]. Local ectopic expression of the *CKX* gene in LRFCs reduces the distance from new neighboring LRP, resulting in increased LR density [9,55,66]. To identify the stage of lateral root development that is sensitive to the inhibitory effects of cytokinin, the *Agrobacterium tumefaciens* cytokinin biosynthesis enzyme isopentenyl-transferase (IPT) was expressed either in XPP cells or young LRPs [9]. Transactivation experiments revealed that XPP cells are sensitive to cytokinin, whereas young lateral root primordia are not [9]. Detailed studies show that cytokinin inhibits the first formative cell division of pericycle founder cells and also continued growth of young LR primordia [67]. In conclusion, these results indicate that cytokinin disrupts the lateral root patterning process by targeting lateral root founder cells. 

An analysis of cytokinin levels in roots suggests that spatiotemporally-regulated metabolic processes contribute to a differential distribution of cytokinin derivatives along the primary root [68]. Specifically, lateral root initiation and primordium formation take place in root zones with high active cytokinin levels; however, lateral root emergence occurs in root zones with an accumulation of biologically-inactive cytokinin conjugates. Moreover, the analysis of the cytokinin response reporter *TCS:GFP* suggests that cytokinin responses are repressed in cells and tissues in which priming and early initiation phases of the lateral root organogenesis take place. Further, mutants affected in cytokinin metabolism and signaling display phenotypes consistent with the possibility that enhanced cytokinin activity in pericycle cells might be important to prevent LRI in close proximity to existing LRP [68]. These results suggest that an enhanced cytokinin response in the pericycle is strongly correlated with LRP initiation.

### 4.2. Cytokinin Suppresses LR Initiation

The spatiotemporal expression pattern of the cytokinin synthesis gene *isopentenyl transferase* (*IPT)* and *lonely guy (LOG)* creates a source of cytokinin in the young primordia, and as a result, establishes an inhibitory field preventing LRI in the neighboring cells [55]. In addition, *IPT3*, *IPT5*, and *IPT7* contribute to cytokinin synthesis in the vicinity of existing LRP, thus suppressing the initiation of new LRs. Consistent with a role for cytokinin in regulating LRP spacing, *ipt3 ipt5 ipt7* and *log4* mutants display aberrant LRP positioning, with LRP found immediately adjacent to existing LRP [55]. The mutation of *CYP735A* genes required for trans-zeatin biosynthesis causes strong defects in LR positioning [55], indicating an important role for the cytokinin metabolite in regulating LR initiation. Further studies show that the expression of a known regulator of LR spacing, the receptor-like kinase Arabidopsis crinkly 4 (ACR4), is reduced in the roots of cytokinin-deficient plants, and the cytokinin status is lowered in *acr4* mutants [55]. In addition, cytokinin and ACR4 inhibit the initiation of neighboring pericycle cells through independent non-hierarchical pathways [55]. Mutants deficient in cytokinin receptors display increased LRP and LR density compared with the wild type [67]. Overall, these studies show that regulated cytokinin synthesis, metabolism, and signaling regulate LR initiation.

### 4.3. Cytokinin Regulates LR Development

*Arabidopsis histidine phosphotransfer protein 6* (*AHP6*) represses cytokinin signaling and is expressed early during lateral root development [11]. The *ahp6* mutant displays defects in the pericycle founder cell divisions that initiate lateral roots [11]. Further, cytokinin treatments result in stage I and stage II LR primordia displaying abnormal pericycle cell divisions [11]. These results are consistent with the possibility that *AHP6*-mediated cytokinin inhibition plays a crucial role in the orientation of cell divisions during lateral root initiation [11]. Thus, in addition to repressing LR initiation, cytokinin affects the lateral root development process.

In summary, cytokinin acts as an important hormonal signal in regulating lateral root initiation and development. In addition, cytokinin directly regulates auxin synthesis, transport, and signaling to influence LR formation and growth on multiple levels by disturbing cell division activity and pattern formation [10,12,55,69,70], as described below.

## 5. Auxin and Cytokinin Act Antogonistically in LR Development

Auxin and cytokinin interact in the control of many central developmental processes in plants, including lateral root development (Figure 2). Classic experiments from Skoog and Miller demonstrated that the balance between auxin and cytokinin is a key regulator of in vitro organogenesis [71]. Exposing callus cultures to a high auxin-to-cytokinin ratio results in root formation, whereas a low ratio of these hormones promotes shoot development [71]. Subsequently, much pharmacological, genetic and transcriptomic evidence confirms the importance of auxin-cytokinin interactions during plant development. In recent years, the existence of synergistic, antagonistic, and additive interactions between these two plant hormones have been uncovered, suggesting a complex web of signal interactions. Moreover, we now know that there is extensive crosstalk between the two hormones at all levels: synthesis, perception, and transport; and we are beginning to understand how these networks interact to control plant lateral root development. 

### 5.1. Auxin-Mediated Regulation of Cytokinin Synthesis and Signaling

Auxin-mediated regulation of cytokinin synthesis was first discovered by treating Arabidopsis roots with auxin, which led to the upregulation of the cytokinin synthesis *IPT5* and *IPT7* genes [72]. Subsequent studies showed that *IPT5:GUS* activity was elevated and its induction was more sensitive to auxin in the *shy2-31* loss-of-function mutant, and *IPT5:GUS* activity was undetectable in the *shy2-2* gain-of-function mutant [73]. Thus, *IPT5* transcript levels are regulated by auxin, mediated through *SHY2*/*IAA3*. In addition to regulating cytokinin biosynthesis, auxin also affects cytokinin degradation by downregulating *CKX2*, *CKX4*, and *CKX7* gene expression and upregulating *CKX1* and *CKX6* in Arabidopsis [74]. In conclusion, auxin affects cytokinin biosynthesis and degradation processes to regulate lateral root initiation. 

Auxin also directly regulates the cytokinin signaling pathway. For example, auxin directly induces the transcription of the type-A *ARR7* and *ARR15* factors through a conserved *cis*-regulatory TGTC motif, thereby suppressing cytokinin output by inhibiting cytokinin signaling transduction in root stem cells [75]. Chromatin immunoprecipitation (ChIP) experiment and electrophoretic mobility shift assay found that ARF5 directly binds to auxin-responsive TGTC elements in the *ARR15* promoter [76]. Taken together, these results suggest that auxin directly regulates the cytokinin signaling pathway.

Overall, auxin plays important roles in regulating cytokinin synthesis and signaling pathways in the context of lateral root development. However, cytokinin also affects auxin biosynthesis and transport pathways. 

### 5.2. Cytokinin-Mediated Modulation of Auxin Metabolism and Transport in LR Development

Cytokinin treatment leads to a rapid increase in auxin biosynthesis in young root and shoot tissues [70]. Reducing endogenous cytokinin levels leads to a reduction in auxin biosynthesis [70]. These results suggest that cytokinin promotes auxin biosynthesis in young root and shoot tissues. Additionally, cytokinin also regulates auxin transport [9,73]. Specifically, cytokinin disrupts the PIN-dependent formation of an auxin maximum during lateral root development [9]. The auxin transcriptional reporter *DR5:GUS* forms a distinct maxima of activity in lateral root primorida. However, this *DR5:GUS* signal is weaker and more diffuse in LRP treated with cytokinin, and the maximum at the primordia tip is often missing [9]. Cytokinin negatively affects *PIN1*, *PIN2*, and *PIN3* transcription, but positively affects *PIN7* [77]. These studies indicate that cytokinin perturbs the pattern of *PIN* gene transcription and the formation of an auxin maximum in LRP. In addition, cytokinin treatment rapidly decreases the plasma membrane *PIN1-GFP* signal in a dose-dependent manner through AHK-based cytokinin perception during LR organogenesis [10]. Indeed, the PIN1 polarity index shifts in favor of anticlinal PIN1 in the *cre1-12*/*ahk4 ahk3* and *cre1-12*/*ahk4 ahk2* mutants during LR primordium formation [12]. Thus, cytokinin is essential to maintaining the basal levels of auxin biosynthesis as well as PIN polarity establishment and redirection of auxin fluxes during lateral root development. 

## 6. Summary

In plants, lateral root formation is critical for the development of root architecture. Physiological and genetic studies have revealed the roles of several plant hormones in lateral root formation, with particularly strong roles for auxin and cytokinin in lateral root initiation and development. In this review, we provide an overview of the lateral development stages (Figure 1) and auxin and cytokinin roles in lateral root development (Figure 2). The interconnections and complexity among various hormones, nutrients, environmental cues, and stress signals involved in lateral root development are only starting to be resolved. Significant challenges remain in understanding the dynamics of these networks. Future research employing computational tools, laboratory experiments and large-scale mutational analyses will be important to forming a deeper understanding of lateral root formation.
